# Antipsychotic Treatment in People at Clinical High Risk for Psychosis: A Narrative Review of Suggestions for Clinical Practice

**DOI:** 10.1097/JCP.0000000000001891

**Published:** 2024-08-05

**Authors:** Alessandro Di Lisi, Simona Pupo, Marco Menchetti, Lorenzo Pelizza

**Affiliations:** From the 1 Department of Biomedical and Neuromotor Sciences, Alma Mater Studiorum Università di Bologna, Bologna; 2 Pain Therapy Service, Department of Medicine and Surgery, Azienda Ospedaliero–Universitaria di Parma, Parma, Italy.

**Keywords:** antipsychotic, clinical high risk, early intervention in psychosis, outcome, psychosis transition, treatment

## Abstract

**Purpose:**

The “early intervention” paradigm in psychiatry holds significant promise for preventing psychosis. Recent evidence showed that individuals at clinical high risk for psychosis (CHR-P) with antipsychotic (AP) prescription at baseline have higher psychosis transition rates compared with those without AP, although the underlying cause remains unclear. In this article, we reviewed international guidelines on early intervention in CHR-P people, paying specific attention to clinical recommendations on AP treatment. Then, we comment on these suggestions in the light of recent empirical evidence examining AP prescription in CHR-P populations within “real-world” clinical settings.

**Methods:**

This search was conducted on PubMed/MEDLINE, PsycINFO, EMBASE, and Google, looking for both “Guidelines AND CHR-P OR UHR OR Early Psychosis.”

**Results:**

International guidelines generally recommend not using AP as first-line treatment, but only when psychosocial interventions have failed. CHR-P people with AP drug showed high prevalence rates and had more severe clinical picture at entry. Is this a “warning signal” for potentially higher psychosis transition risk? Is it a direct AP iatrogenic effect? Is it possible to detect specific CHR-P subgroup that may benefit from AP? These are the questions that this article seeks to explore.

**Conclusions:**

The current framework for identifying CHR-P subjects has defined psychometric criteria mainly based on positive symptoms. In our opinion, this is reductive, especially for evaluating therapeutic outcomes and prognosis. A more comprehensive assessment considering quality of life, psychiatric comorbidity, persistent negative symptoms, subjective experience of CHR-P psychopathology, and social/personal recovery is thus needed.

In medicine, prevention remains a global health care priority. In mental health, McGorry^1^ pioneered preventive interventions in early psychosis, defining a staging model mainly focused on clinical high risk for psychosis (CHR-P) mental states with the aim of grasping the psychotic disorder's developmental trajectories and identifying high-risk individuals early. Indeed, first-episode psychosis (FEP) patients typically had earlier, attenuated psychotic symptoms (APSs) up to 5–10 years before developing their full-blown psychosis.^2^ Early detection/intervention on CHR-P individuals should thus avoid psychosis transition, reduce the duration of untreated psychosis, and improve prognosis and clinical outcomes.^3^

Currently, there are 2 main approaches to detect CHR-P individuals: (*a*) “ultrahigh-risk” (UHR) criteria and (*b*) “basic symptoms” (BS) criteria.

According to McGorry,^1^ 3 *UHR* subgroups were defined: (*a*) APSs; (*b*) brief, limited, intermittent psychotic symptoms; and (c) genetic risk and functioning deterioration (GRFD) syndrome (see Fig. 1 for details). These UHR subgroups may be reliably assessed using specific clinical interviews, such as the Comprehensive Assessment of At-Risk Mental States (CAARMS).^4,5^

**FIGURE 1:**
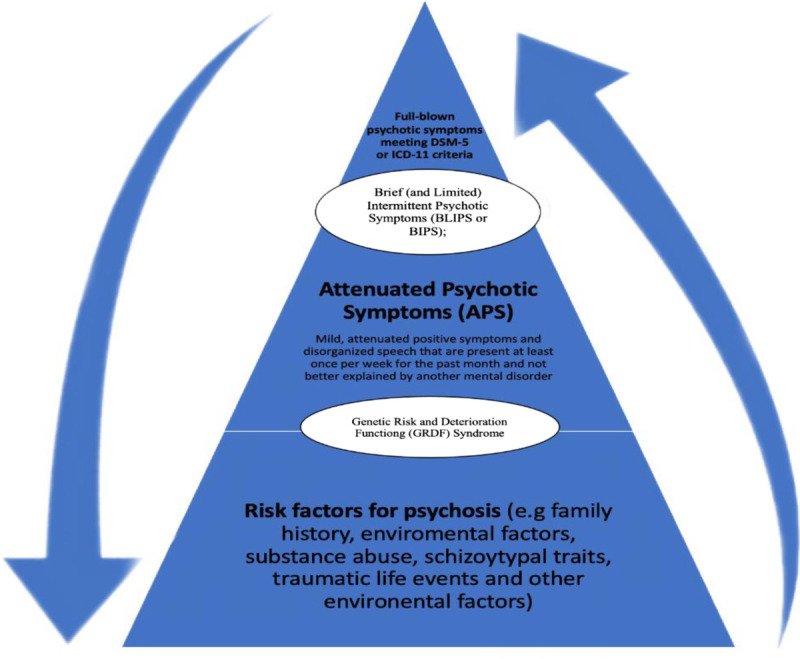
Early course of psychosis. The APS subgroup includes most UHR subjects, with more than 80% prevalence rates in different studies. APS individuals have mild, attenuated positive symptoms and/or disorganized speech that must be of sufficient severity or frequency to warrant clinical attention, must have been present at least once per week for the past month, and must have begun or worsened in the last year. They are also not better explained by other mental disorders (including affective disorders with psychotic features) and are not attributable to the physiological effects of a substance or another medical condition. Finally, criteria for any psychotic disorder have never been met. BLIPS individuals meet the criteria for psychosis for less than a week, and their psychotic symptoms cease spontaneously, without AP medication. The GRFD syndrome is a state-trait condition combining a family history of psychosis in first-degree relatives or schizotypal personality disorder in the individual with 30% drop in functioning or chronic low functioning in the past year. BLIPS, brief, limited, intermittent psychotic symptoms; *DSM-5*, *Diagnostic and Statistical Manual of Mental Disorders, 5th Edition*; GFRD, genetic risk and functioning deterioration; *ICD-11*, *International Classification of Diseases, 11th Revision*.

The alternative/integrative approach for identifying CHR-P individuals, especially those at clinical high risk for schizophrenia, is known as the “BS model”^6^ and specifically investigates the presence of subtle, self-experienced disturbances in affective, perceptual, and cognitive processes that may anticipate and transit to full-blown psychotic symptoms (especially the positive ones) (see Fig. 2 for details). Years of research and evidence reported that specific cognitive and perceptual BS was shown to effectively predict clinical high-risk individuals who will subsequently transit to overt psychosis.^7^ However, these BSs are not confined to the prodromal stage of psychosis, but may also occur during and after an acute psychotic episode.^8^ Reliable assessment of BS includes the administration of the Schizophrenia Proneness Instrument, Adult, or Child and Youth version.^9^

**FIGURE 2:**
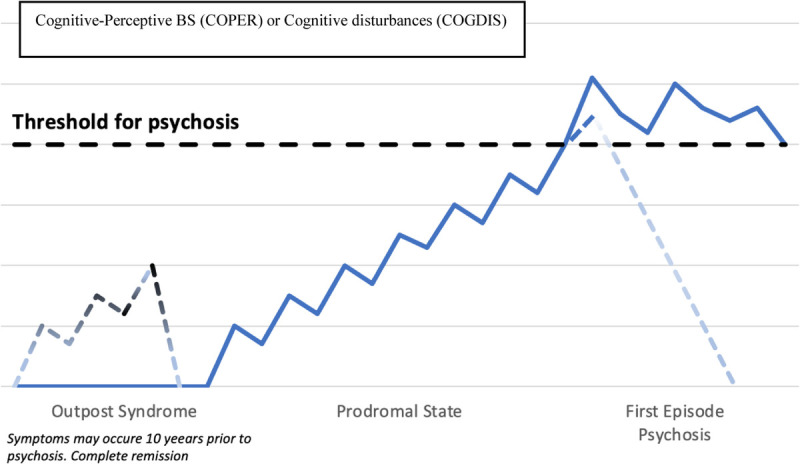
The Basic Symptom (BS) model. COPER included the following 10 cognitive and perceptual BS: thought interference, thought blockages, disturbance of receptive speech, thought pressure, unstable ideas of references, thought perseveration, decrease ability to discriminate between ideas/perception or fantasy/true memory, derealization, visual perception disturbances, and acoustic perception disturbances. COGDIS included the following nine cognitive basic symptoms: inability to divide attention, thought interference, thought blockages, disturbance of receptive speech, disturbance of expressive speech, thought pressure, unstable ideas of references, disturbances of abstract thinking, and captivation of attention by details on the visual field.

## Antipsychotic Medication in CHR-P Mental States

The prescription of antipsychotic (AP) medication in young people at CHR-P is a delicate, complex, and often divisive issue, where official guidelines and real-world prescription habits seldom correspond, especially in adolescence.^10^ In this respect, AP use in CHR-P individuals is relatively common in clinical practice, with reported baseline prevalence rates ranging from 23% to 77% in different studies.^11^ This is in ostensible tension (if not in open contradiction) with current treatment guidelines for CHR-P, recommending a more cautious approach and generally excluding AP drugs as first-choice therapy.

In CHR-P populations, the most likely factors influencing clinicians' decision-making on AP prescription are related to global functioning decline and clinical severity of positive/disorganized symptoms.^12,13^ It has been also reported that AP prescription in CHR-P subjects is mainly intended at delaying psychosis transition, reducing patient's psychological distress, and improving clinical outcomes.^14^ However, to date, empirical evidence for actual benefits of AP therapy in preventing psychosis and improving long-term outcomes in CHR-P individuals is still conflicting, and international guidelines' recommendations on early psychosis treatment partially differ from each other, with some experts against primarily using AP drugs while others being more optimistic (particularly in adults with severe psychopathology and relevant functioning decline).^15^

This narrative review mainly aimed at comparing international guidelines' recommendations on AP prescription in CHR-P people to update mental health professionals on an ongoing debated topic and encourage prescribing practices aligned with expert advice and evidence-based information. Given recent empirical advances on AP use in CHR-P populations, in our opinion there is a need to bridge the gap between the cautious prescription approach recommended by international guidelines and the usual “real-world” clinical practice.^16^

## MATERIALS AND METHODS

In this article, we reviewed all international guidelines on early intervention in CHR-P people published and available in the following search engines: PubMed/MEDLINE, PsycINFO, EMBASE, and Google (Table 1). In particular, a combination of the following key terms was applied (in the “title/abstract” field): “Guidelines” OR “Recommendation” AND “CHR-P” OR “UHR” OR “Early Psychosis.” We considered guidelines exclusively written in English or in Italian and excluded guidelines not specifically discussing CHR-P intervention, guidelines published more than 10 years ago, or unpublished guidelines.

**TABLE 1:** International Guidelines' Recommendations on AP Prescription in CHR-P

	Year	First-Line Treatment	AP Medication	
NICE	2016	CBT	Do not offer AP medication	
EPA	2015	CBT	Use low-dose second-generation AP if psychological treatment has proved ineffective or CHR-P symptoms get worse	
ACG	2016	CBT	AP only if there is evidence of at least 1 week of frank positive psychotic symptoms, or if positive symptoms are milder or briefer, but are directly associated with risk of self-harm or aggression	Omega-3 fatty acids
CTG	2017	Individual CBT with or without family intervention; treat comorbid disorders	Use low-dose second-generation AP for short-term period only if previous psychological interventions have proven ineffective	
Emilia-Romagna recommendations (Italy)	2023	CBT	AP only if functional impairment, a high risk of self-healing, and ineffectiveness of first-line psychosocial interventions	

NICE, National Institute for Health and Care Excellence; CTG, Canadian Treatment Guidelines.

## RESULTS

Out of a total of 8 guidelines identified for consideration, only 5 were included in this review because they specifically dealt with the topic of our interest. Two guidelines were excluded because of addressing early intervention in FEP or schizophrenia^17,18^ and one because it was published in 2005.^19^

The “National Institute for Health and Care Excellence” guidelines on recognition and management of psychosis and schizophrenia in children and young people^20^ recommend psychological intervention (specifically cognitive-behavioral therapy [CBT]) as first-line treatment. They explicitly indicate not to offer AP medication in any case.

The European Psychiatric Association (EPA) guidance on early intervention in CHR states of psychoses^21^ suggests that in adults it should be applied the least restrictive service approach, using CBT as first-line choice. Where psychotherapy has shown to be ineffective, it should be complemented by low-dose second-generation AP drug (especially if severe and progressive CHR-P symptoms occur), with the primary purpose to reach symptom stabilization. Any long-term AP treatment with preventive goal is not recommended. The EPA also suggests that any intervention in CHR individuals should also address current individual needs and other comorbid mental problems (such as depression and anxiety).

According to the EPA guidelines, although CBT and pharmacological interventions are helpful to prevent or at least postpone an FEP in adult CHR-P subjects, evidence on the psychosis predictive value of CHR criteria and effectiveness of psychosocial and psychopharmacological treatments in CHR-P children and adolescents is still not sufficient to justify primarily preventive interventions in these young populations. In children and adolescents, the EPA suggests that specific psychological therapies aimed at improving functioning should be provided as part of an overall intervention plan and complemented by treatments for other psychosocial problems and comorbid mental disorders. Indeed, an early intervention in individuals at CHR-P should aim not only at preventing FEP, but also the development/persistence of functional (social/educational/vocational) deficits.

The Australian Clinical Guidelines (ACG) for early psychosis^22^ recommend CBT as first-choice intervention for young people at CHR-P. CBT is primarily advised to alleviate APS, so as to potentially delay/prevent psychosis transition. Individual psychotherapy and supportive counseling may also enhance preonset social functioning.

According to the ACG, AP medication is avoided unless a week of full-blown positive symptoms occurs. Exception includes brief or mild positive symptoms associated with self-harm or high aggression risk, substance-related psychosis, or subthreshold psychotic symptoms persisting despite CBT and/or other interventions and causing distress or disability. The ACG also indicates that omega-3 fatty acids may aid in delaying/preventing psychosis conversion in CHR-P people.

The Canadian Treatment Guidelines for individuals at CHR-P^23^ recommend offering individual CBT (with/without family intervention) as first-line therapy in the least restrictive approach. If psychotherapy is ineffective and severe, progressive APSs persist, low-dose second-generation AP medication can be added but only for CHR-P adults. However, long-term preventive AP treatment is discouraged.

As the only case in Italy, the Emilia-Romagna Working Group on early intervention in young people at CHR-P or with FEP^24^ recommends against prescribing AP drug for subthreshold psychotic symptoms and/or for reducing psychosis transition risk. However, exclusively in CHR-P adults, a careful clinical assessment for AP prescription should take into consideration the presence of accelerated functioning impairment, a high risk of self-harm behaviors, and ineffectiveness of first-line psychosocial interventions. In any case, AP medication should be prescribed for a short time and primarily aimed at alleviating psychological distress.

## DISCUSSION

In general, international guidelines align in prioritizing individual psychotherapy (CBT) as first-line approach for managing CHR-P mental states. Integrating social support interventions and family psychoeducation also enhances long-term outcomes in CHR-P people.^25^ Conversely, the use of AP medication for preventive purposes remains incompletely resolved. On the one hand, there are some guidelines advocating short-term AP prescription to mitigate CHR-P symptoms and to reduce the risk of transition to full-blown psychotic features (particularly when psychosocial treatments are insufficient or ineffective), although specifics about AP prescribing duration are still quite undefined. Differently, other guidelines recommend against AP use in CHR-P populations, also due to their potential side effects (such as weight gain and sexual dysfunction) and self-stigmatization.

Therefore, although CBT is widely endorsed, the role of AP medication remains debated, inducing a careful case-by-case consideration of its potential risks and benefits.^26^ Anyway, a critical question arises: regardless of the recommendations of international guidelines, what is the “real” diffusion of AP prescription in the common clinical practice?

### AP Prescription, “Real-World” Clinical Setting, and the Risk of Psychosis Transition

In the CHR-P research paradigm, baseline AP exposure is too often neglected, despite its crucial impact on initial psychopathology, clinical outcomes, and psychosis transition risk evaluation.^27^ In this respect, many prognostic models for transdiagnostic staging and individualized risk stratification in psychosis are based on a combination of multiple baseline parameters, but disregard other potentially relevant outcome modulators, such as ongoing APs or other pharmacological therapies.^28^ Indeed, the clinical habit of prescribing AP medication in CHR-P people with the main aim of both symptom stabilization and preventing/delaying psychosis transition should not be underestimated.^29^

Recent studies specifically examining baseline AP exposure in CHR-P individuals showed relevant AP prescription rates, ranging from 23% in meta-analytic research^11^ to 51% and 77% in Italian and Chinese CHR-P samples, respectively.^27,30^

Moreover, cumulative evidence paradoxically reported that CHR-P people with baseline AP exposure (AP+) show higher incidence rates of psychosis transition compared with CHR-P individuals without AP (AP−) across different follow-ups, independently from age, gender ratio, overall sample size, sample enrichment, follow-up length, and quality of the studies.^31,32^ Specifically, AP+ subjects have an imminent increase in their risk of psychosis transition, whereas AP− reached a plateau of their risk between 24 and 36 months.^33^ Among AP+ individuals, monotherapy and/or low-dose AP treatment also seem to be associated with lower transition rates.^34^

Based on these unexpected findings, some crucial questions arise. How should we consider AP treatment not effective and harmful in attenuated clinical pictures and at the same time beneficial (especially for positive symptoms) in more severe ones (eg, FEP)? Why AP therapy should work with difficulty in CHR-P people experiencing similar but attenuated psychotic features, so as not to prevent psychosis conversion?

Raballo et al^35^ solved this paradox inverting the order of causality, suggesting that AP+ subjects have higher psychosis transition rates because mental health clinicians, in “real-world” settings, frequently recommend AP medication to individuals with severe CHR-P symptoms at the enrollment in specialized services. AP prescription would be a “need-based” secondary option motivated by the perception of increasing severity by the treating staff. Albeit apparently arbitrary and subjective, the decision to prescribe APs is generally shared with patients, clinicians, and caregivers and reflects a global apprehension of the severity of clinical status (ie, that of a mental state requiring nondeferable AP therapy). Such increasing severity in psychopathology experienced by AP+ individuals (compared with AP−) could plausibly enhance the risk of symptomatically overt transition to psychosis and to develop full-blown psychotic features despite the pharmacological prescription. If this is the case, the AP prescription pattern rather than an iatrogenic harm factor (inducing conversion to psychosis in CHR-P subjects) could be better conceptualized as a severity indicator of the ongoing psychopathological process (ie, the more severe the process, the less likely to obtain symptom stabilization with AP low doses and monotherapy). The discrepancy may not solely reflect the clinical severity of symptoms, which often necessitates immediate pharmacological intervention, but also broader challenges including limited access to alternative treatments, clinician preferences, systemic pressures, and the expectations of patients and caregivers.

Alternatively, other authors traced this epidemiological finding to a direct iatrogenic effect of AP drugs. In this respect, van Os and Kapur^36^ suggested that psychotic symptoms could be caused by an excessive synthesis/release in presynaptic dopamine. In CHR-P individuals, AP medication could increase the risk of supersensitivity states blocking D_2_ postsynaptic receptors and leading to a D_2_-receptor upregulation.^37^ These supersensitivity states could thus explain why AP+ subjects have higher transition rates to psychosis as an iatrogenic effect of AP medication. Despite the need for evidence using PET procedures to establish the presence of a D_2_ receptor upregulation, Bastiampillai and colleagues^37^ suggested that clinicians should always be cautious in prescribing AP drug to CHR people, especially in childhood and adolescence.

Based on these findings, Raballo et al^31^ suggested that we could better enrich the short-term prognostic staging of CHR-P status with clinically pragmatic anchor points along a gradient of progressive need of care (parallel to the risk of incurring in severe outcomes): AP-naive CHR-P, CHR-P in low-dose AP monotherapy, CHR-P in high-dose AP monotherapy, or AP polytherapy. According to the authors, this classification would avoid the “clinical optic illusion” that AP-exposed CHR-P subjects presenting APSs have the same prognostic risk of conversion to psychosis than AP-naive ones. Indeed, ongoing AP therapy in young CHR-P people could possibly mitigate the initial presentation of their clinical picture and modulate the later outcome trajectories, thereby blurring predictive modeling and prognostic estimates. CHR-P individuals with baseline AP exposure might surreptitiously be equated to all other AP-naive CHR-P subjects, while they actually experienced an AP-attenuated FEP.^33^ In contrast, CHR-P with incident AP therapy at baseline may not reach the formal psychometric threshold for psychosis at follow-up (because of the ongoing treatment), yet their ascription to the “nonconverter” subgroup together with AP-naive CHR-P subjects is highly questionable.^31^ In this sense, AP treatment may be considered as a systematically overlooked confounder whose magnitude in mainstream CHR-P literature clearly influences our current prognostic estimates of longitudinal outcomes and reduces the precision of contemporary prediction models in early psychosis.

Furthermore, it is also important to consider neurocognitive assessment. Indeed, a recent systematic review highlighted substantial neurocognitive deficits in CHR-P individuals when compared with control subjects and also interestingly with FEP patients. These deficits are spanning a wide range of cognitive domains: visual memory, verbal learning skills, and count. Such insights are not only crucial for early detection and intervention strategies, but also hold significant prognostic value. They suggest that specific neurocognitive deficits could help to identify individuals at elevated risk of transitioning to psychosis, potentially pinpointing those who may derive the most benefit from AP prescriptions.^38^ Also, a noteworthy biomarker in this context is the diminished amplitude of “mismatch negativity” (MMN), which could be a potential indicator for the likelihood and timing of psychosis conversion, particularly in CHR-P subjects not yet treated with AP medication. Moreover, the utility of MMN as a neurophysiological target opens up novel avenues for treatment development within CHR-P populations, particularly strategies focusing on modulating NMDA-receptor glutamate transmission, given NMDA receptors' essential role in mediating MMN responses.^39^

Finally, CHR-P people with analog symptom profiles (eg, similar CAARMS positive, negative, and general scores) but with or without ongoing AP treatment cannot be ascribed to the same “a priori” risk class. This might actually help take a further step toward precision psychiatry.^40^ Indeed, within the general “red flag” conferred by AP prescription, high-dose therapy and/or polytherapy are proxies of an ever higher psychopathological risk (ie, of mental states, which would be insufficiently stabilized with lower doses or monotherapy).

### AP Prescription in CHR-P Individuals: Suggestions From Clinical Practice

Prescribing AP drug in CHR-P people is never an easy choice, although in “real-world” clinical practice, it seems to represent more frequently a pondered decision on a rational, need-based clinical evaluation. In this sense, such decision threshold appears to be primarily motivated by the identification of a global mental state (not always limited to positive symptoms) in urgent need of AP treatment. This condition is clearly a nonnegligible index of clinical severity, also crucial for psychopathological risk management.^41^

Despite international guidelines agreement on the fact that AP medication should not be used as first-line treatment (also because this potentially exposes young CHR-P individuals to various side effects without certain clinical benefits),^42^ mental health care clinicians seem to follow the common clinical sense and are more likely to prescribe AP medication to those subjects who have greater severity levels of CHR psychopathology and poorer socio-occupational functioning.

Furthermore, although no clear evidence on higher effectiveness of any current preventive treatment in avoiding/delaying the risk of psychosis transition was reported,^43^ in our opinion a definitive judgment on the clinical utility of AP prescription in CHR-P population cannot be based on the risk of psychosis conversion as main outcome. In general, the literature in this field overfocused symptom-based criteria for transition to psychosis (ie, specific quantitative threshold exclusively based on positive symptoms scores as mapped by the CAARMS), without considering other relevant clinical, social, and personal characteristics and outcomes, and neglecting the original Australian criteria on “functional equivalents” of such transition (ie, the threshold at which AP treatment would probably be commenced in common clinical practice).^44^

In our opinion, this current psychometric supremacy of positive symptoms makes the CHR-P population clinically indefinite and too heterogeneous. Mental health clinicians and researchers should make an effort to identify specific CHR-P subgroups with different functioning and clinical outcomes, using also neurocognitive assessment and biomarker like diminished amplitude of MMN, because these subgroups might respond differently to AP treatment. Indeed, exclusively using psychometric (positive symptom-based) criteria for identifying CHR-P mental states and psychosis conversion is not enough to assess the actual effectiveness of AP treatment. It is essential to consider other relevant clinical variables beyond positive psychopathology and psychosis conversion, such as real-world/socio-occupational performance, the presence of psychiatric comorbidity (including substance use disorder and past traumatic events), persistent negative symptoms, longitudinal diagnostic trajectories, subjective sense of well-being, personal/social recovery, inclusion in the local community, and individual experience of patient's own lived world. In this respect, if 2 CHR-P individuals with the same psychometric score on the CAARMS have different subjective experiences in terms of quality of life, personal recovery, and meaning of their psychological suffering, they also could differ in terms of potential effectiveness of all interventions provided, as well as in the risk of psychosis conversion. It is also important to consider that APs have demonstrated efficacy in managing APSs, contributing significantly to the acute treatment phase.^45,46^ Nevertheless, the long-term implications of AP use on functional recovery and quality of life remain unclear. Notably, APs, particularly at high doses or in polypharmacy, are associated with substantial adverse effects encompassing metabolic, neurological, and cognitive domains. These side effects may exacerbate functional impairment or necessitate hospitalization due to medication-related complications. For instance, sedation and weight gain could worsen the individual's capacity for daily activities or discourage return to work or school. Moreover, the psychosocial ramifications of AP prescription, such as stigma or the psychological impact of being labeled as requiring psychiatric medication, may further affect clinical and functional outcomes. In the light of these side effects, establishing a trust-based relationship with patients and caregivers and adopting a patient-centered perspective with emphasis on shared decision-making are essential, also considering that these individuals are young.^47^

Using correlation analysis, Zhang and colleagues^48^ categorized CHR-P individuals into different psychopathological subgroups and found the highest psychosis conversion risk in those subjects with persistent negative symptoms and cognitive deficits. Therefore, employing different tools for a more comprehensive evaluation of CHR-P individuals can enhance our understanding of CHR-P mental states beyond positive psychopathology, broadening empirical research outcomes and informing treatment effectiveness assessment. Zhang et al^49^ also highlighted different prognostic trajectories for CHR-P subtypes, indicating potential prediction of treatment response. By using a personal risk assessment, the authors confirmed restricting AP prescription to CHR subjects with predominant positive symptoms and functional decline. This insight advocates a stringent AP prescription strategy to curtail inappropriate use.

## CONCLUSIONS

The results of this narrative review suggest that there is no convincing evidence on AP prescription in CHR-P people. Therefore, the choice of whether prescribing AP medication in CHR-P individuals rests with mental health clinicians, patients, and their family members. However, this does not seem an entirely arbitrary choice, but it appears to take into consideration the current global clinical condition of CHR-P subjects, going beyond the positive psychometric criteria currently defined for categorizing CHR-P mental states.

In this sense, mental health professionals usually seem to meet and talk in depth with each patient, empathize with individual suffering, and give importance not only to symptom severity, but also to other relevant personal, social, and functioning variables. Therefore, according to Raballo et al,^11^ the clinician's judgment about AP prescription in CHR-P people may be considered as an index of clinical severity and, in pragmatic terms, a warning signal (a severity “red flag”) for potentially higher incipient risk of psychosis than expected on the bases of the crude positive symptom score, so designating a “hyper–CHR-P" subgroup as compared with AP-naive CHR-P. The current guidelines for AP prescription in CHR-P have remained static for over 7 years, underscoring an urgent need for updates to reflect recent research findings and clinical advancements. A more nuanced approach, such as identifying specific subgroups within the CHR-P population, could reveal individuals more likely to benefit from AP therapy. Furthermore, executing large-scale trials similar to the EUFEST^50^ or the CATIE studies,^51^ but specifically tailored to CHR-P cohorts, would furnish more accurate data on the efficacy of AP treatments, including critical metrics such as the “number needed to treat” and the “number needed to harm.”^52^

In our opinion, for better evaluating the effectiveness of interventions (including AP treatment), a comprehensive assessment of CHR-P individuals could be helpful, also based on the following relevant clinical variables: real-world performance, persistent negative symptoms, longitudinal diagnostic trajectories, social/work/school functioning, subjective experience of psychological suffering, quality of life, psychiatric comorbidities, personal and social recovery, stigma, inclusion in the local community, neurocognitive assessment, and diminished amplitude of MMN.^47^ At the same time, this could allow us to broaden outcome parameters in CHR-P research beyond transition to psychosis and to identify specific CHR-P subgroups with potentially different prognosis and response to AP drug. However, some questions remain open: Who are these CHR-P subjects and how can we reliably define them? When to start AP treatment for psychotic symptoms: at the premorbid phase or at the FEP onset? To date, it is far-fetched to believe that people at CHR-P may be effectively categorized exclusively by what their positive psychopathological manifestations are. Future studies examining which different CHR-P subgroups might have benefits from AP prescription are thus absolutely needed.
